# LncRNA NEAT1 promotes the tumorigenesis of colorectal cancer by sponging miR‐193a‐3p

**DOI:** 10.1111/cpr.12526

**Published:** 2018-11-08

**Authors:** Hong‐Mei Yu, Chen Wang, Zhen Yuan, Guang‐Liang Chen, Tao Ye, Bi‐Wei Yang

**Affiliations:** ^1^ Department of Oncology, Zhongshan Hospital Fudan University Shanghai China; ^2^ Liver Cancer Institute, Zhongshan Hospital Fudan University Shanghai China

**Keywords:** colorectal cancer, growth, LncRNA NEAT1, miR‐193a‐3p

## Abstract

**Objectives:**

LncRNA nuclear‐enriched abundant transcript 1 (NEAT1) participates in the development and progression of multiple malignancies. However, the molecular mechanism by which NEAT1 contributes to colorectal cancer (CRC) remains unclear.

**Methods:**

The association between lncRNA NEAT1 expression and clinicopathological characteristics and prognosis in patients with CRC was analysed by TCGA RNA‐sequencing data. MTT, colony formation, flow cytometry, transwell assays and a xenograft tumour model were used to assess the functions of NEAT1. Bioinformatics and spearman correlation analysis were used to identify the NEAT1‐specific binding with miRNAs, and luciferase gene report and RIP assays were performed to confirm the interaction between miR‐193a‐3p (miR‐193a) and NEAT1 in CRC cells.

**Results:**

Upregulation of NEAT1 expression was significantly correlated with TNM stage, poor survival and tumour recurrence in patients with CRC, and acted as an independent prognostic factor for tumour recurrence. Knockdown of NEAT1 suppressed cell proliferation, colony formation abilities and invasive potential and induced cell apoptosis, but overexpression of NEAT1 reversed these effects. Furthermore, NEAT1 was confirmed to act as a sponge of miR‐193a, and knockdown of NEAT1 attenuated miR‐193a inhibitor‐induced tumour promoting effects and L17RD expression in CRC cells. miR‐193a harboured negative correlation with NEAT1 and IL17RD expression in CRC specimens. In vivo experiment further validated the inhibitory effects of NEAT1 knockdown on xenograft tumour growth.

**Conclusion:**

Our findings demonstrate that lncRNA NEAT1 acts as an oncogenic role in CRC cells by sponging miR‐193a and may represent a potential marker for CRC patients.

## 1 INTRODUCTION

Colorectal cancer (CRC) is one of the most common malignant diseases worldwide.^1^ Approximately 1‐2 million patients are diagnosed with CRC, and more than 0.6 million die from this disease.^2^ Although colorectal tumours at an early stage can be removed by surgical and endoscopic resection, more than 50% of CRC patients are found at an advanced stage and have poor survival and recurrence due to tumour invasiveness.^3^ Colorectal tumorigenesis is caused by various factors in the complicated multi‐stage process involving the successive accumulation of genetic alterations. Thus, identification of these potential biomarkers is indispensable for early detection and diagnosis of CRC.

Long non‐coding RNAs (lncRNAs) are a class of endogenous non‐coding RNAs of more than 200 nucleotides and have no capability to encode the functional proteins. But, increasing evidence shows that they act a role in gene expression and regulation, RNA processing and translation in human diseases including cancer.^4^, ^5^ NEAT1 as a nuclear‐restricted lncRNA was thought to promote myeloid differentiation in acute promyelocytic leukaemia^6^ and androgen receptor‐associated prostate cancer progression.^7^ Subsequently, NEAT is responsible for reducing chemotherapy sensitivity^8^, ^9^ and accelerating tumorigenesis in breast cancer,^10^ ovarian cancer^11^ and bladder cancer,^12^ acting as a potential prognostic predictor of glioma.^13^

Accumulating data indicate that lncRNAs act as competing endogenous RNAs (ceRNAs) to reduce the activity of microRNAs (miRNAs) through shared miRNA response elements (MREs) in cancer.^14^, ^15^ For example, NEAT1 facilitates tumour progression in lung cancer,^16^ and laryngeal squamous cell carcinoma by miR‐107/CDK6 axis^17^ and in pancreatic cancer by miR‐335/c‐met axis,^18^ and contributes to the chemo‐resistance to gemcitabine in cholangiocarcinoma.^19^ These studies unveil the key regulation crosstalk between NEAT1 and miRNAs in cancer.

Although NEAT1 was previously reported to serve as a marker for CRC,^20^ the functions of NEAT1 in CRC are still unknown. In this study, we found that NEAT1 expression was upregulated in CRC samples and was associated with TNM stage and poor prognosis, acting as an independent prognostic factor of tumour recurrence in patients with CRC. Moreover, NEAT1 promoted the tumorigenesis of CRC cells by sponging miR‐193a and represented a potential marker for CRC patients.

## 2 MATERIALS AND METHODS

### 2.1 Materials

CRC cell lines (LOVO, HCT116, DLD‐1, Caco2 and SW480) and normal tissues used in our study were from Liver Cancer Institute of Zhongshan Hospital. Lentivirus‐mediated sh‐NEAT1 or negative control (NC) vectors, virion‐packaging elements, miR‐193a mimic and inhibitor were purchased from Genechem (Shanghai, China); The antibodies against E‐cadherin (24E10, rabbit monoclonal antibody), N‐cadherin (#4061, rabbit polyclonal antibody), Vimentin (D21H3, rabbit monoclonal antibody) and PCNA (#13110, rabbit monoclonal antibody) were from Cell Signaling Technologies (Beverly, MA, USA) and anti‐IL17RD (PA5‐21682, rabbit polyclonal antibody) was from Thermo Fisher Scientific (Waltham, MA, USA). The TCGA RNA sequencing data of CRC patients were downloaded from the website (https://xenabrowser.net/heatmap/) and summarized in Table S1.

### 2.2 Drugs and reagents

Dulbecco's modified Eagle's medium (DMEM) and foetal bovine serum (FBS) were from Thermo Fisher Scientific Inc (Waltham, MA, USA); MTT was from Sigma‐Aldrich (St. Louis, MO, USA); TRIzol Reagent was from Invitrogen (Carlsbad, CA, USA); M‐MLV Reverse Transcriptase was from Promega (Madison, WI, USA); SYBR Green Master Mixture was from Takara (Otsu, Japan); ECL‐PLUS/Kit was from GE Healthcare (Piscataway, NJ, USA).

### 2.3 Plasmid construction

We commercially synthesized the miR‐193a inhibitor and mimic (100 nmol/L), and the wild type NEAT1 vector, which contained the miR‐193a binding sites, and the mutant fragment, which contained the mutant binding sites of miR‐193s, were obtained by annealing double‐strand DNA and inserted into the pmirGLO vector at the BamHI and EcoRI sites. The full‐length NEAT1 (accession number: NR_028272) was amplified with the following primers: forward, 5′CTTCCTCCCTTTAACTTATCCATTCAC‐3′; reverse, 5′‐ CTCTTCCTCCACCAT TACCAACAATAC‐3′. Then, it was cloned into the EcoRI and MluI sites of the pCMV‐GFP vector. The sh‐NEAT1 plasmid, expressing a siRNA that targets NEAT1 transcription, was constructed by annealing single‐strand hairpin cDNA and the detailed description referred to the reference.^18^

### 2.4 Cell culture and lentiviral transfection

CRC cells were cultured in DMEM medium supplemented with 10% heat‐inactivated FBS, 100 U/mL of penicillin and 100 μg/mL of streptomycin. Cells in this medium were placed in a humidified atmosphere containing 5% CO2 at 37°C. When cells reached 60% confluence, they were transfected with recombinant experimental virus or control virus, and cultured at 37°C and 5% CO2 for 4 hours. Then supernatant was discarded and serum containing growth medium was added. Positive and stable transfectants were selected and expanded for further study.

### 2.5 Quantitative Real‐time PCR (qRT‐PCR)

To quantitatively confirm the mRNA expression levels of NEAT1 in CRC cell lines and tissues, real‐time PCR was performed. Total RNA was extracted from each clone using TRIzol according to the manufacturer's protocol. Reverse transcription was carried out using M‐MLV and cDNA amplification was performed using the SYBR Green Master Mix kit according to the manufacturer's guidelines. GAPDH or U6 gene was used as an endogenous control. A miScript Primer Assay (QIAGEN) was used for the miR‐193a and U6. Data were analysed using the comparative Ct method (2−^△△^ *^C^* ^t^). Three separate experiments were performed for each clone. The primers used were listed in Table S2.

### 2.6 Western blot analysis

CRC cell lines were harvested and extracted using lysis buffer (Tris‐HCl, SDS, Mercaptoethanol, Glycerol). Cell extracts were boiled for 5 minutes in loading buffer, and then, equal amount of cell extracts were separated on 12% SDS‐PAGE gels. Separated protein bands were transferred into polyvinylidene fluoride (PVDF) membranes. The primary antibodies against E‐cadherin, N‐cadherin, Vimentin, IL17RD and PCNA were diluted according to the instructions of antibodies and incubated overnight at 4℃. Then, horseradish peroxidase‐linked secondary antibodies were added at a dilution ratio of 1:1000, and incubated at room temperature for 2 hours. The membranes were washed with PBS, and the immunoreactive bands were visualized using ECL‐PLUS/Kit according to the kit's instruction. The relative protein level in different groups was normalized to GAPDH concentration. Three separate experiments were performed for each clone.

### 2.7 Cell viability assay

Cell proliferation was analysed by the MTT assay. CRC cells were incubated in 96‐well plates at a density of 2 × 10^3^ cells per well with DMEM medium supplemented with 10% FBS. Cells were treated with 20 μL of MTT and subsequently incubated with 150 μL of DMSO for 15 min. The colour reaction was measured at 570 nm using an Enzyme Immunoassay Analyzer (Bio‐Rad, Hercules, CA, USA).

### 2.8 Colony formation assay

2× DMEM containing 20% FBS and 2 × 10^3^ cells was mixed with equal volume of 0.7% agarose and immediately plated in 6‐well plates containing an underlayer of 0.5% agarose made in 1× DMEM supplemented with 10% FBS. The plates were cultured at 37°C under 5% CO2 for 10 days.

### 2.9 Cell invasion and apoptosis assays

Cell transwell assay and flow cytometry analysis were conducted for assessing the cell invasive potential and apoptotic index as previously reported.^18^

### 2.10 Dual‐luciferase reporter assay

CRC cells were seeded into 24‐well plates. After 24‐hours incubation, pmirGLO report vector carrying wild type or mutated of NEAT1 was co‐transfected with miR‐193a mimic (100 nmol/L) or miR‐NC into the LOVO and HCT116 cells. Forty‐eight hours after transfection, luciferase activities were examined with a Dual‐luciferase Reporter System (Promega).

### 2.11 Animal experiments

Six‐week‐old female immune‐deficient nude mice (BALB/c‐nu) were bred at the laboratory animal facility. All experimental procedures were performed according to the regulations and internal biosafety and bioethics guidelines of Zhongshan Hospital, Fudan University. Mice were injected subcutaneously with 5 × 10^6^ LOVO CRC cells. Mice were monitored daily and developed a subcutaneous tumour. The tumour volume every three days was measured with a calliper using the formula: volume = (length × width)2/2. The expression levels of lncRNA NEAT1 and miR‐193a were detected between sh‐NEAT1 group and sh‐NC group by qRT‐PCR analysis.

### 2.12 Statistical analysis

SPSS 18.0 (IBM, SPSS, Chicago, IL, USA) was used for the statistical analysis. All of the values were recorded as the Mean ± SEM from at least three independent experiments. Two‐tailed Student's *t* test was used to evaluate the differences between each group. The cut‐off value of NEAT1 was determined by the NEAT1 expression level, survival time and survival status (the number of death n = 94 or survivors n = 298) and it was used to distinguish the NEAT1 high expression (n = 60) or low expression level (n = 332), by which survival curves were plotted using the Kaplan‐Meier method and were assessed for the statistical significance using a log‐rank test. Statistical significance was set at *P* < 0.05.

## 3 RESULTS

### 3.1 LncRNA NEAT1 expression was upregulated in human CRC samples

**Figure 1:**
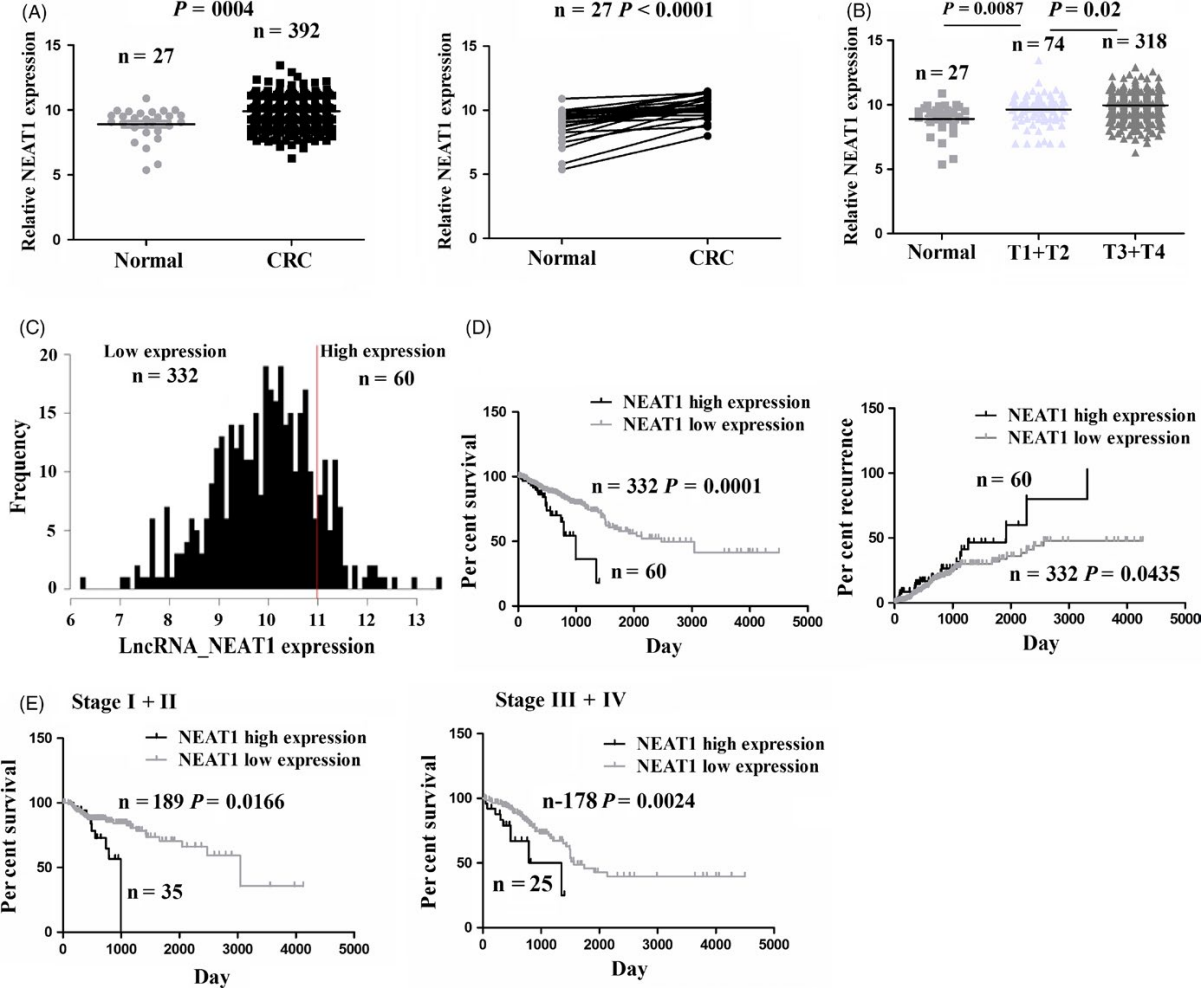
LncRNA NEAT1 was upregulated in CRC tissues and associated with poor survival and recurrence. A, TCGA RNA sequencing data analysis of the expression of NEAT1 in CRC (n = 392) and adjacent normal (n = 27) as well as in paired CRC (n = 27). B, The expression of NEAT1 in CRC patients with T1+T2 stage (n = 74) or T3+T4 stage (n = 318) and normal tissues (n = 27). C, The expression of NEAT1 was divided into high expression (n = 60) or low expression group (n = 332) according to the cut‐off value in CRC. D, The correlation of NEAT1 high expression or low expression with overall survival and recurrence of CRC patients. E, The correlation of NEAT1 high expression or low expression with overall survival of CRC patients with early stage or late stage

Some studies have shown that lncRNA NEAT1 expression is increased in some cancers.^10^, ^11^, ^12^, ^13^ To verify the expression of NEAT1 in CRC tissues, we used 2015 TCGA sequencing data (https://genome-cancer.ucsc.edu/), which showed that NEAT1 expression was significantly upregulated in unpaired (n = 392) or paired CRC samples (n = 27) as compared to the normal tissues (n = 27) (Figure 1A). In addition, NEAT1 expression was elevated in CRC with T3+T4 stage (n = 318) as compared to those with T1+T2 stage (n = 74) or normal tissues (n = 27) (Figure 1B).

### 3.2 LncRNA NEAT1 expression was correlated with poor survival and recurrence in patients with CRC

We further analysed the correlation of NEAT1 expression with the clinicopathological features and prognosis in patients with CRC. Based on the NEAT1 expression level, overall survival (OS) time and survival status, we obtained a suitable cut‐off value of NEAT1 in 392 CRC patients (Figure S1A) using the cut‐off finder (https://molpath.charite.de/cutoff/load.jsp), among which NEAT1 was divided into high expression group and low expression group (Figure 1C). As shown in Table 1, NEAT1 high expression was positively associated with TNM stage (*P = *0.024), but had no correlation with age, gender, tumour localization, pathological stage and lymphatic invasion of the patients (each *P* > 0.05). We then drew the survival and recurrence curves, which showed that the CRC patients with NEAT1 high expression had shorter survival and higher tumour recurrence as compared to those with NEAT1 low expression (Figure 1D). Moreover, the patients of early stage or late stage with NEAT1 high expression had the shorter survival (Figure 1E), but had no difference in tumour recurrence as compared to those with NEAT low expression (Figure S1B).

**Table 1:** The correlation of NEAT1 expression with clinicopathological characteristics of CRC patients

Variables	Cases (n)	NEAT1		*P* value
		High	Low	
Total	392	60	332	
Age (years)				
≥60	256	36	220	
<60	136	24	112	0.349
Gender				
Male	211	31	180	
Female	181	29	152	0.716
Pathological stage				
I/II	224	37	187	
III/IV	168	23	145	0.442
Localization				
Colon	299	42	257	
Rectum	93	18	75	0.329
TNM stage				
T1+T2	74	5	69	
T3+T4	318	55	263	0.024
Lymphatic invasion				
Negative	285	47	238	
Positive	107	13	94	0.288

In addition, univariate cox regression analysis revealed that NEAT1 high expression was related with an increased risk of the survival (RR 1.299, 95% CI 0.974 to 1.500; *P* = 0.025) and recurrence of CRC (RR 1.246, 95% CI 1.001 to 1.550; *P* = 0.042) (Tables S3 and S4). Considering all the potential confounding factors, multivariate Cox regression analysis showed that NEAT1 expression was an independent predictor of tumour recurrence (Table S4) in patients with CRC.

### 3.3 LncRNA NEAT1 promoted cell growth and reduced cell apoptosis

**Figure 2:**
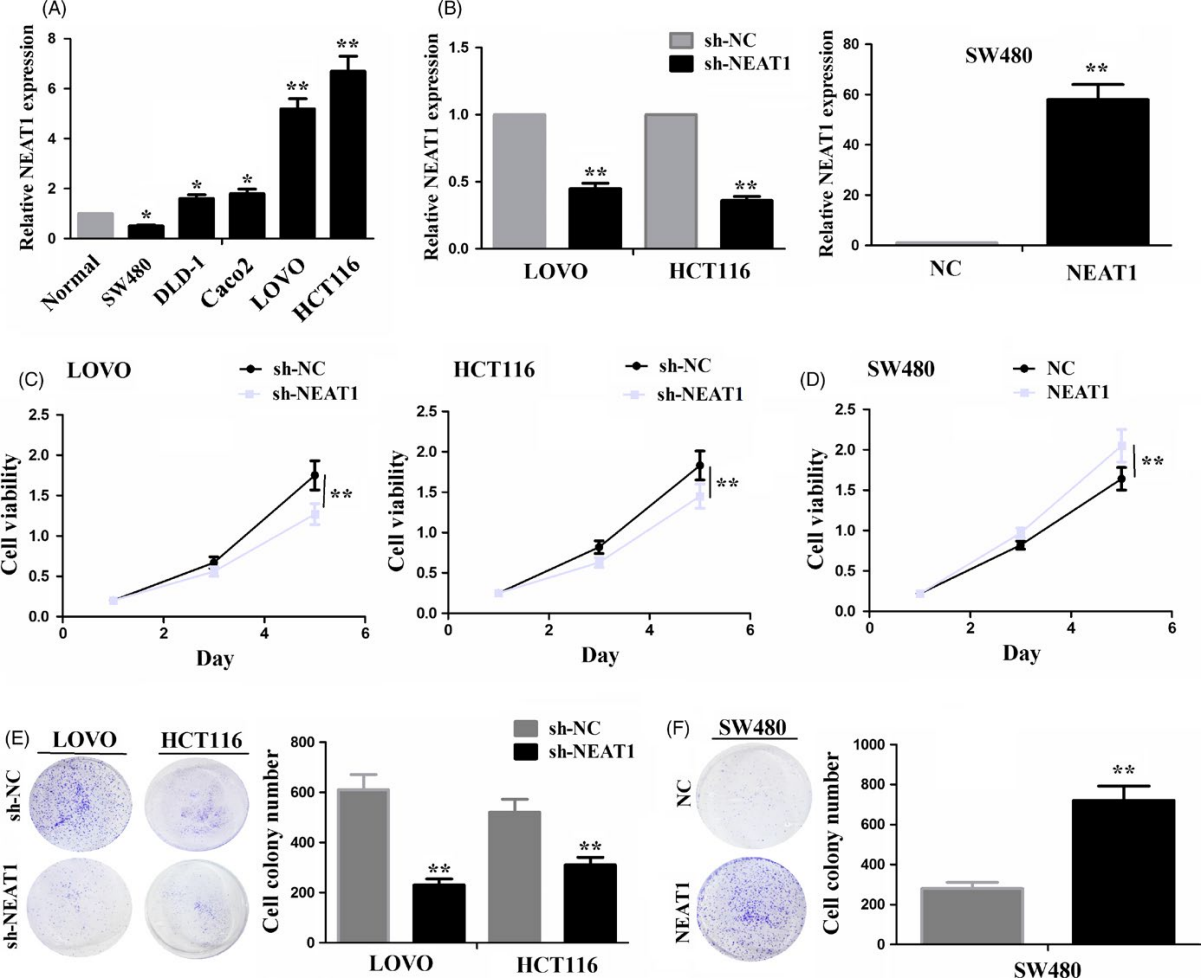
LncRNA NEAT1 promoted CRC cell growth. A, qRT‐PCR analysis of the expression levels of NEAT1 in different CRC cell lines. B, The knockdown or overexpression efficiency after transfection with sh‐NEAT1 in LOVO and HCT116 cell lines or NEAT1 in SW480 cell line indicated by qRT‐PCR analysis. C, MTT assessment of cell proliferation viability in LOVO and HCT116 cells after transfection with sh‐NEAT1 or sh‐NC. D, MTT evaluation of cell proliferation viability in SW480 cells after transfection with NEAT1 or NC. E, Effects of NEAT1 knockdown on cell colony formation in LOVO and HCT116 cells. F, Effects of NEAT1 overexpression on cell colony formation in SW480 cells. * *P < *0.05; ** *P < *0.01

Increased expression of NEAT1 in CRC tissues indicated its tumour‐promoting role in CRC. To validate this hypothesis, we examined the expression level of NEAT1 in different CRC cell lines, indicating that it had lower expression in SW480 cell line but higher expression in LOVO and HCT116 cell lines as compared to the colon normal tissue (Figure 2A). Then, the knockdown efficiency of sh‐NEAT1 in LOVO and HCT116 cell lines or overexpression efficiency of NEAT1 in SW480 cell line was identified by qRT‐PCR analysis (Figure 2B). Then, knockdown of NEAT1 decreased cell viability in LOVO and HCT116 cell lines as compared to the sh‐NC vector (Figure 2C), but ectopic expression of NEAT1 displayed a proliferation promoting effect in SW480 cells (Figure 2D). In addition, the number of colony formation in sh‐NEAT1 transfected LOVO and HCT116 cell lines was significantly reduced as compared to empty vector (Figure 2E), but NEAT1 overexpression increased the colony formation number in SW480 cell line (Figure 2F). Interestingly, we also assessed the effects of NEAT1 on cell apoptosis in CRC cells by flow cytometry analysis, indicating that the index of cell apoptosis in sh‐NEAT1 transfected LOVO and HCT116 cell lines was markedly increased as compared to sh‐NC group (Figure S2A), but NEAT1 overexpression decreased the cell apoptosis in SW480 cell line (Figure S2B).

### 3.4 LncRNA NEAT1 promoted CRC cell invasion

**Figure 3:**
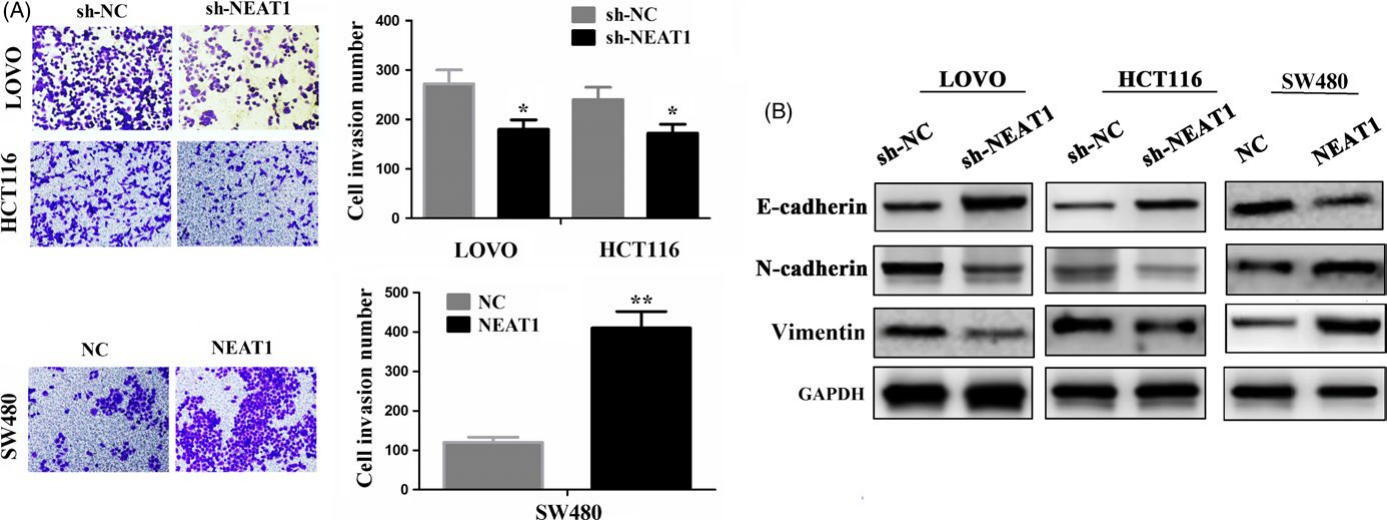
LncRNA NEAT1 promoted invasion of CRC cells. A, Cell invasion ability was determined by transwell in CRC cells after transfection with sh‐NEAT1 or NEAT1 vectors. B, The protein expression levels of EMT markers (E‐cadherin, N‐cadherin and Vimentin) were detected by Western blot after transfection with sh‐NEAT1 in LOVO and HCT116 cell lines or NEAT1 in SW480 cell line. * *P < *0.05; ** *P < *0.01

To observe the effects of NEAT1 on CRC cell invasion, we conducted a transwell invasion assay, which showed that knockdown of NEAT1 weakened cell invasive potential in LOVO and HCT116 cell lines, but overexpression of NEAT1 promoted these effects in SW480 cell line (Figure 3A). The protein expression of epithelial‐mesenchymal transition (EMT) markers, such as E‐cadherin, N‐cadherin and Vimentin, was detected by Western blotting analysis (Figure 3B), indicating that knockdown of NEAT1 increased the protein levels of E‐cadherin, but decreased N‐cadherin and Vimentin expression in LOVO and HCT116 cell lines. Inversely, overexpression of NEAT1 decreased E‐cadherin expression but increased N‐cadherin and Vimentin expression in SW480 cell lines.

### 3.5 LncRNA NEAT1 acted as a sponge of miR‐193a in CRC cells

**Figure 4:**
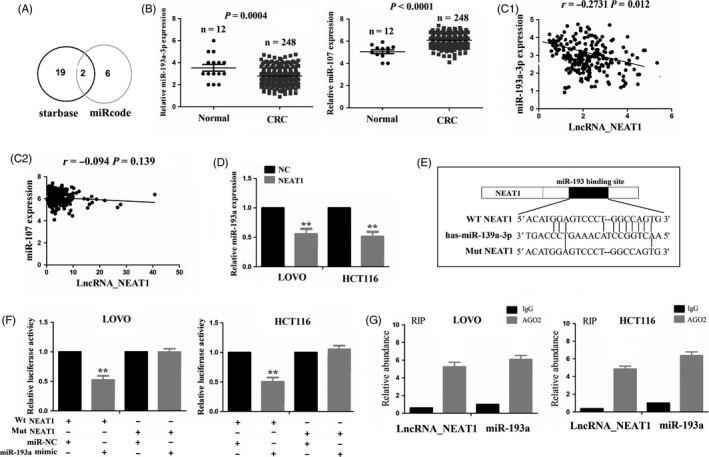
LncRNA NEAT1 acted as a sponge for miR‐193a in CRC cells. A, Two miRNAs‐miR‐107 and miR‐193a‐3p were simultaneously identified to bind with lncRNA NEAT1 by starBase v 2.0 and miRcoed. B, TCGA RNA sequencing data analysis of the expression level of miR‐193a and miR‐107 in CRC samples. C, TCGA RNA sequencing data analysis of the correlation of NEAT1 expression with miR‐107 and miR‐193a‐3p expression in CRC samples. D, qRT‐PCR analysis of the expression of miR‐193a after transfection with NEAT1 overexpression in LOVO and HCT116 cell lines. E, The binding sequences of miR‐193a and wt or Mut NEAT1. F, The luciferase activity of wt or Mut NEAT1 after co‐transfection with miR‐193a mimic and wt or mut NEAT1 reporter vector. G, RIP assay analysis of the interaction of NEAT1 and miR‐193a with Ago2 protein. ** *P < *0.01

To uncover the molecular mechanism by which NEAT1 contributes to CRC, using the bioinformatics analysis software starBase v2.0 (https://starbase.sysu.edu.cn/targetSite.php), according to the very high stringency (>5) and its expression in more than three tumour styles, 23 miRNAs were identified to bind with NEAT1 (Table S5). Meanwhile, using miRcode (https://www.mircode.org/index.php), eight miRNAs were found to interact with NEAT1 (Table S6). Thus, miR‐107 and miR‐193a‐3p (miR‐193a) were simultaneously identified to have the potential to bind with NEAT1 by starBase v2.0 and miRcode (Figure 4A). Furthermore, miR‐193a was downregulated but miR‐107 was upregulated in CRC samples as compared to normal tissues (Figure 4B), and NEAT1 had a negative correlation with miR‐193a expression (Figure 4C1), but had no correlation with miR‐107 expression (Figure 4C2) in CRC tissues. NEAT1 reduced the expression of miR‐193a (Figure 4D), but miR‐193a had no effect on the expression of NEAT1 (Figure S3) in LOVO and HCT116 cell lines, indicated by qRT‐PCR analysis. The binding sites of miR‐193a with wide type (WT) or mutant (Mut) NEAT1 are indicated in Figure 4E. To further confirm whether NEAT1 was a target of miR‐193a, we co‐transfected LOVO and HCT116 cells with WT or Mut NEAT1 reporter vector and the miR‐193a mimic or miR‐NC, indicating that miR‐193a mimic decreased the luciferase activity of WT NEAT1 (Figure 4F).

Previous studies have shown that miRNAs act as miRNA ribonucleoprotein complexes including Ago2, an important component of RNA‐induced silencing complex (RISC).^21^ Given that Ago2 generally interacts with RNAs in the cytoplasm, we then conducted a RIP assay using anti‐Ago2 antibody. Both NEAT1 and miR‐193a were enriched by five to ‐ sixfold following immunoprecipitation using the anti‐Ago2 antibody as compared to anti‐IgG in LOVO and HCT116 cell lines (Figure 4G).

### 3.6 NEAT1 knockdown counteracted miR‐193a inhibitor‐induced tumour promoting effects in CRC cells

**Figure 5:**
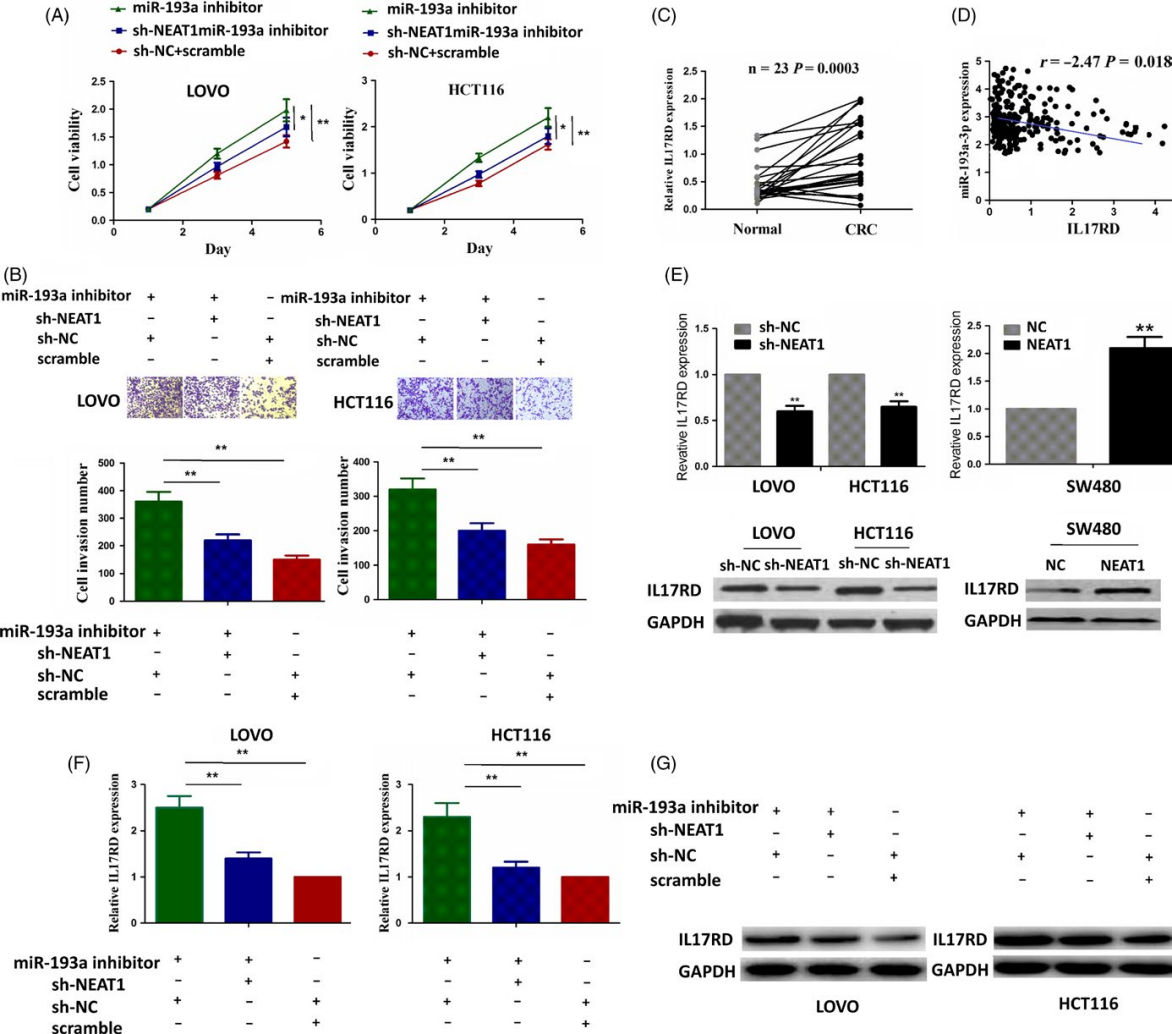
NEAT1 knockdown counteracted the tumour promoting effects of miR‐193a inhibitor in CRC cells. A, B, MTT and cell colony formation assays showed that miR‐193a inhibitor increased cell proliferative activity and colony formation capacity, but these tumour promoting effects induced by miR‐193a were attenuated by NEAT1 knockdown in LOVO and HCT116 cell lines. C, TCGA data analysis of the expression level of IL17RD in pared CRC samples. D, TCGA data analysis of the correlation of IL17RD expression with miR‐193a in CRC samples. E, qRT‐PCR and Western blot analysis of the effects of lncRNA NEAT1 on the expression of IL17RD. F, G, qRT‐PCR and Western blot analysis showed that miR‐193a inhibitor increased the expression of IL17RD and this effect was reversed by NEAT1 knockdown in LOVO and HCT116 cell lines. * *P < *0.05; ** *P < *0.01

We detected the miR‐193a expression level in LOVO and HCT116 cell lines after transfection with miR‐193a inhibitor or Scramble by qRT‐PCR (Figure S4). To understand the molecular mechanisms by which miR‐193a mediates the functions of NEAT1 in CRC cells, miR‐193a inhibitor and sh‐NEAT1 were co‐transfected into LOVO and HCT116 cell lines, indicating that miR‐193a inhibitor promoted cell proliferation and invasive potential, but knockdown of NEAT1 counteracted these tumour promoting effects induced by miR‐193a inhibitor (Figure 5A,B). Previous studies showed that IL17RD was a direct target of miR‐193a in CRC cells.^22^ We also identified IL17RD as a key target of miR‐193a according to the cumulative weighted context score (−0.95) using the TargetScan (https://www.targetscan.org/vert_71/) (Table S7). TCGA data showed that IL17RD expression was markedly upregulated in CRC (Figure 5C) and had the negative correlation with miR‐193a expression in CRC (Figure 5D). To uncover the direct relationship between NEAT1 and IL17RD, we transfected the shNEAT1 into LOVO and HCT116 CRC cell lines and NEAT1 overexpression vector into SW480 cell lines, and qRT‐PCR and Western blot analysis (Figure 5E) showed that knockdown of NEAT1 downregulated the expression of IL17RD and overexpression of NEAT1 upregulated IL17RD expression. Furthermore we investigated the effects of co‐transfection of miR‐193a inhibitor and sh‐NEAT1 on the expression of IL1RD by qRT‐PCR (Figure 5F) and Western blot assays (Figure 5G), which demonstrated that miR‐193a inhibitor increased the expression of IL17RD but knockdown of NEAT1 attenuated this increased effect induced by miR‐193a inhibitor.

### 3.7 Knockdown of NEAT1 inhibited xenograft tumour growth

**Figure 6:**
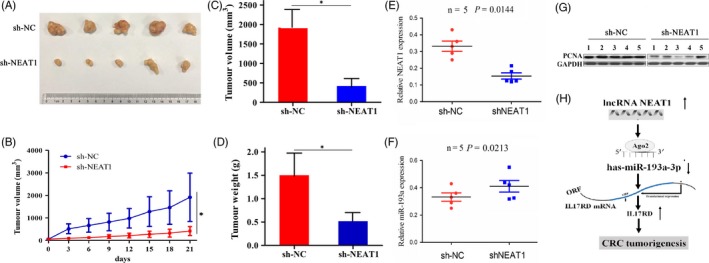
Knockdown of NEAT1 inhibited xenograft tumour growth. A, Representative photographs of xenograft tumour after treatment with sh‐NEAT1 or sh‐NC. B, Growth curves of tumour proliferation after treatment with sh‐NEAT1. C, D, The average volumes and weight in sh‐NEAT1 and sh‐NC groups after treatment for 3 weeks. E, F, qRT‐PCR analysis of the expression levels of NEAT1 and miR‐193a in xenograft tumour tissues after treatment with sh‐NEAT1 or sh‐NC. G, Western blot analysis of the expression level of PCNA in xenograft tumour tissues after treatment with sh‐NEAT1 or sh‐NC. H, LncRNA NEAT1 could sponge miR‐193a, thereby increasing IL17RD expression and promoting CRC tumorigenesis. *P < 0.05

Having investigated the tumour promoting effects of NEAT1 on CRC cells in vitro*,* we further checked its effect in vivo. A subcutaneous LOVO xenograft model was established to observe the tumour growth activity affected by NEAT1 knockdown. During the tumour growth period, the growth activity of xenograft tumour was measured. We found that the proliferation rates of tumours were lowered by NEAT1 knockdown (Figure 6A,B). When the tumours were harvested, the average volumes and weight in sh‐NEAT1 group were decreased compared with sh‐NC group (Figure 6C,D). Then, we extracted the RNA and protein from the tumour tissues derived from the sh‐NEAT1 and sh‐NC groups, and detected the expression levels of NEAT1 and miR‐193a by qRT‐PCR and that of PCNA by Western blot analysis, which indicated that NEAT1 expression was decreased (Figure 6E), but miR‐193a expression was increased (Figure 6F) in sh‐NEAT1 group as compared to sh‐NC, and knockdown of NEAT1 downregulated the expression of PCNA in tumour tissues compared with the sh‐NC group (Figure 6G).

## 4 DISCUSSION

LncRNA NEAT1 is implicated in diverse biological processes and acts as a potential predictor for survival and recurrence in CRC,^20^ gastric cancer (GC)^23^ and oesophageal squamous cell carcinoma.^24^ Herein, we found that NEAT1 was upregulated in CRC, and associated with tumour stage, overall survival and recurrence, acting as an independent prognostic factor for tumour recurrence in patients with CRC. Our findings corroborated the previous studies in CRC^20^ and GC.^23^ These studies suggest that NEAT1 might act as a potential biomarker in CRC.

Functionally, our studies showed that overexpression of NEAT1 markedly enhanced the growth and invasion of CRC cells, while knockdown of NEAT1 impaired these effects in vitro. Using the xenograft tumour model, we evidenced that knockdown of NEAT1 inhibited CRC tumour growth in vivo. PCNA is a key indicator of tumour proliferation. We also found that NEAT1 knockdown reduced the PCNA expression level in xenograft tumour model. These results were supported by the previous findings in other cancers ,^12^, ^16^, ^17^, ^18^, ^19^, ^23^, ^24^, ^25^, ^26^, ^27^ which uniformly revealed the tumour promoting role of NEAT1 in cancer. Moreover, lncRNAs act as miRNA sponges to promote tumour progression. For example, lncRNA H19 mediates breast cancer metastasis by sponging miR‐200b/c and let‐7b,^28^ lncRNA UCC accelerates CRC progression by sponging miR‐143.^29^ NEAT1 also sponges miR‐449b‐5p/c‐Met axis to promote glioma pathogenesis.^26^ In this study, we confirmed miR‐193a‐specific binding with NEAT1 by luciferase assay and validated a negative correlation between NEAT1 expression and miR‐193a in CRC samples. Moreover, NEAT1 overexpression reduced the expression level of miR‐193a in CRC cell lines and miR‐193a expression was increased in tumour tissues derived from sh‐NEAT1 group as compared with the sh‐NC group. RIP assay showed that NEAT1 and miR‐193a could bind to Ago2 protein, suggesting NEAT1 might function as a sponge of miR‐193a in CRC.

It is known that miR‐193a acts as a potential tumour suppressor in malignant tumours.^30^ On the one hand, it inhibits tumour metastasis in osteosarcoma by targeting Rab27B and SRR^31^ and in lung cancer by targeting ERBB4/PIK3R3/mTOR/S6K2 pathway.^32^ On the other hand, miRNA‐193a enhances cell migration in prostate cancer by targeting AJUBA^33^ and promotes multi‐chemoresistance in bladder cancer by targeting HOXC9^34^ and LOXL4.^35^ Consistent with the previous study,^22^ we here found that miR‐193a was downregulated in CRC samples and had the negative correlation with IL17RD expression, which has been confirmed as a direct target of miR‐193a in CRC.^22^ Then, miR‐193a inhibitor increased proliferation and invasion and upregulated IL17RD expression in CRC cells, and these tumour promoting effects induced by miR‐193a inhibitor were counteracted by NEAT1 knockdown. NEAT1 also upregulated the expression of IL17RD, but knockdown of NEAT1 downregulated its expression. Our results inferred that NEAT1 might act as a sponge of miR‐193a to reduce its activity, and increase IL17RD expression, resulting in CRC tumorigenesis. (Figure 6H).

## 5 CONCLUSIONS

In conclusion, our findings demonstrated that lncRNA NEAT1 promoted CRC progression via sponging miR‐193a and was associated with poor survival and recurrence of CRC patients. Our study might provide an intriguing biomarker for CRC patients.

## CONFLICTS OF INTEREST

The authors declare that they have no competing interests.
