# Supplementary figures and images for: LncRNA NEAT1 promotes the tumorigenesis of colorectal cancer by sponging miR‐193a‐3p
Source: Cell Prolif. 2018 Nov 8;52(1):e12526. doi: 10.1111/cpr.12526 (PMC6430453; doi:10.1111/cpr.12526)

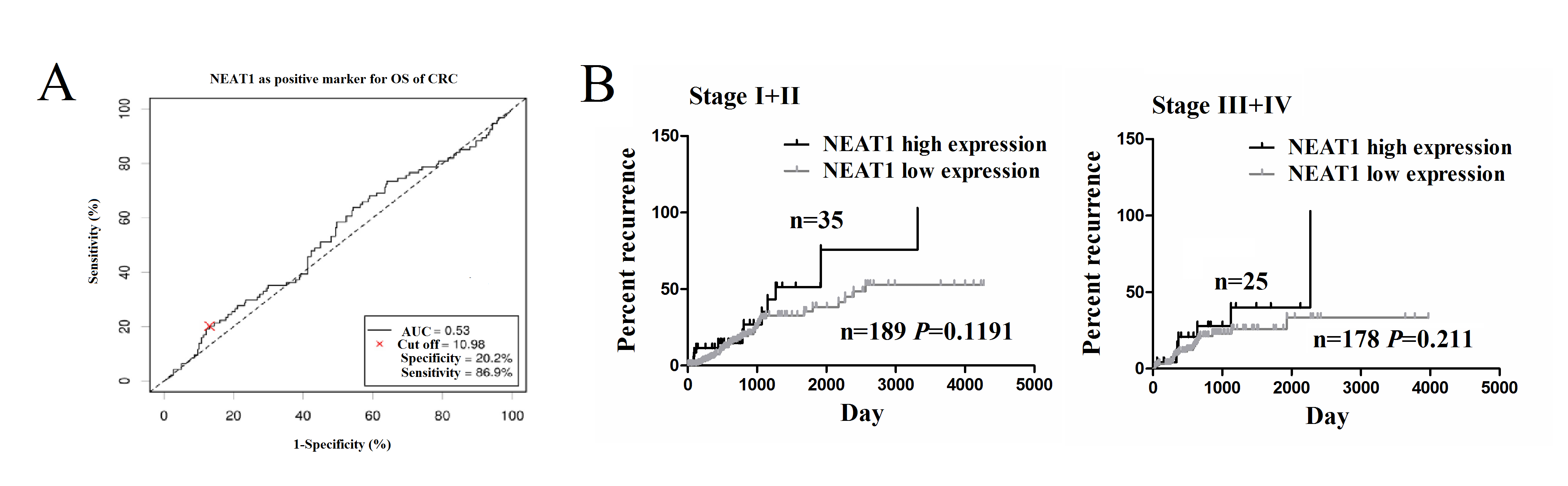

Supplement: Supplementary file 1 [file CPR-52-e12526-s001.tif]

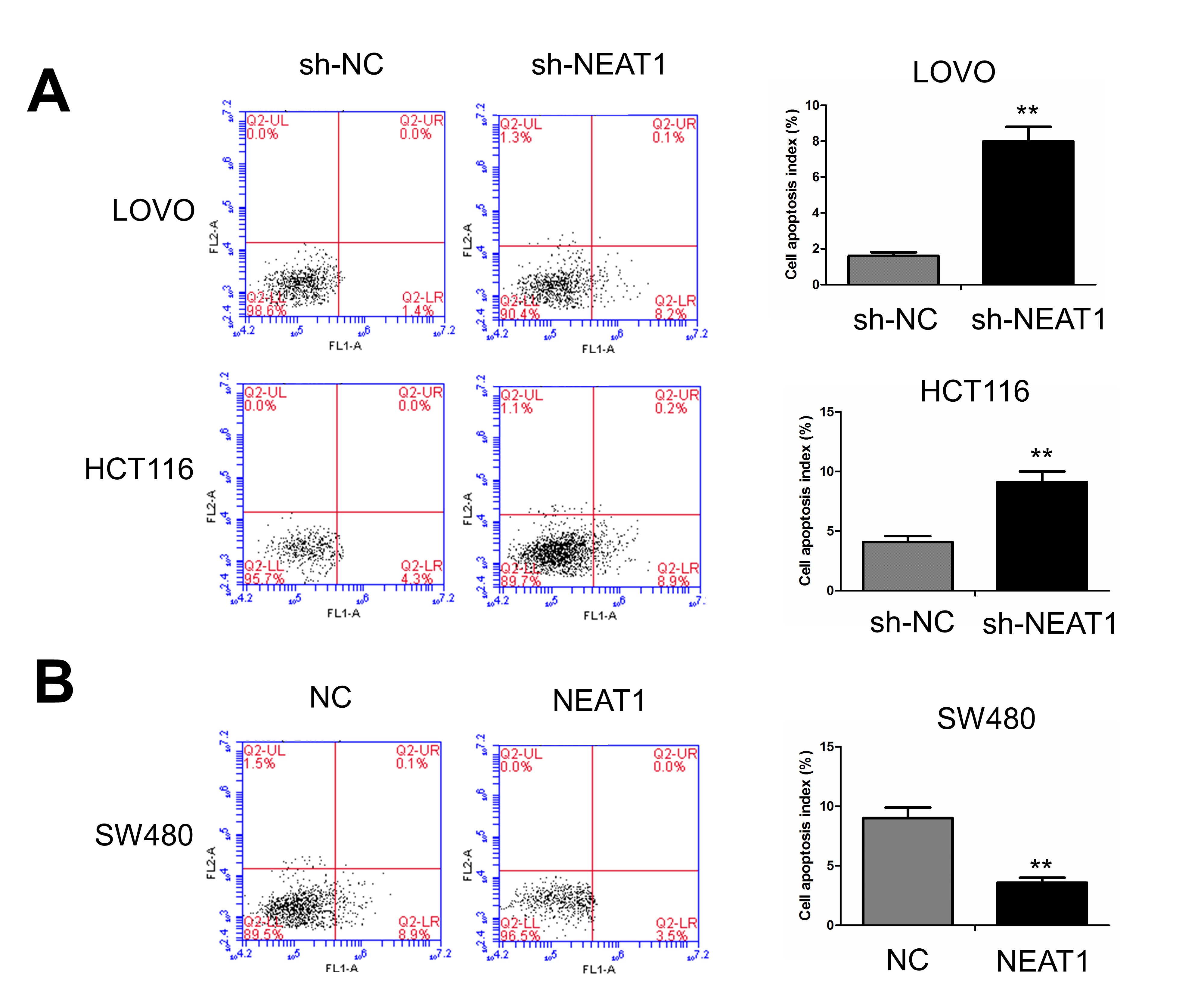

Supplement: Supplementary file 2 [file CPR-52-e12526-s002.tif]

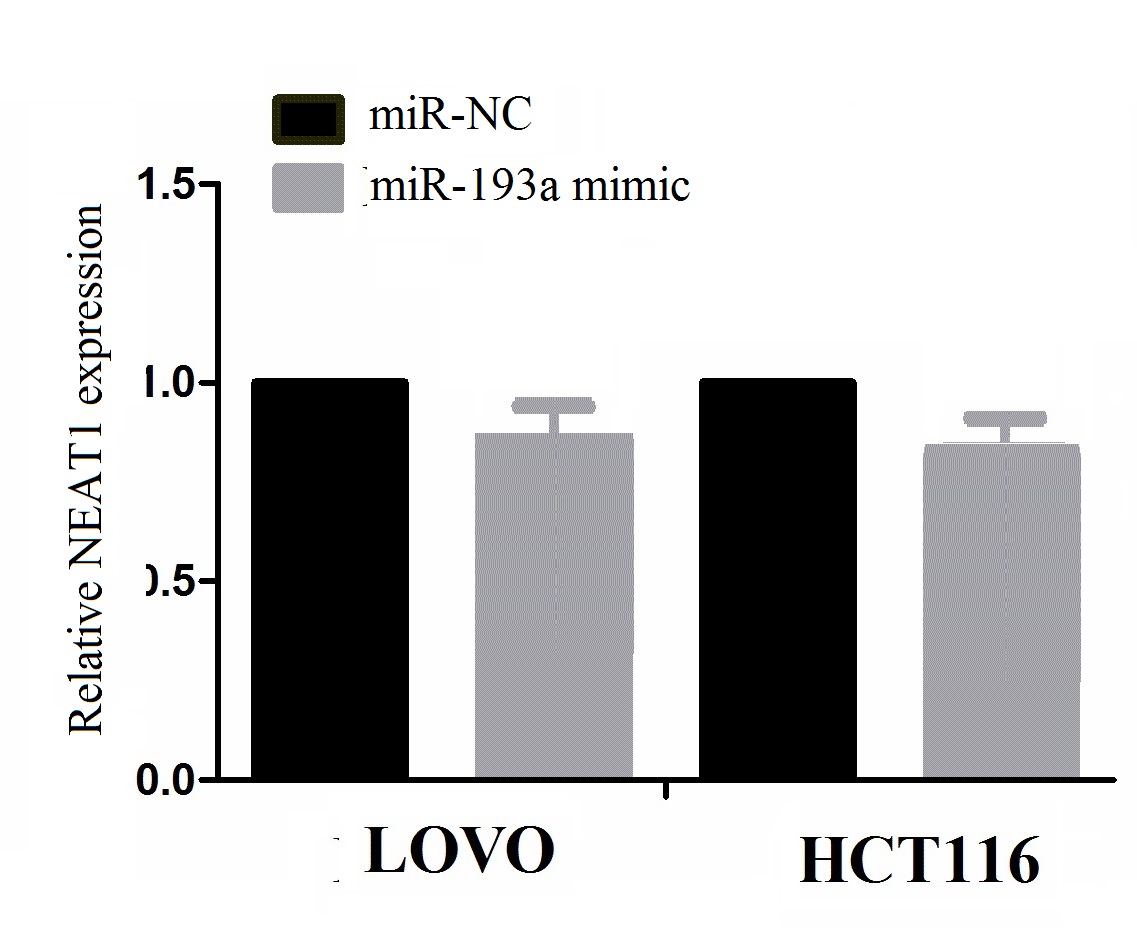

Supplement: Supplementary file 3 [file CPR-52-e12526-s003.jpg]

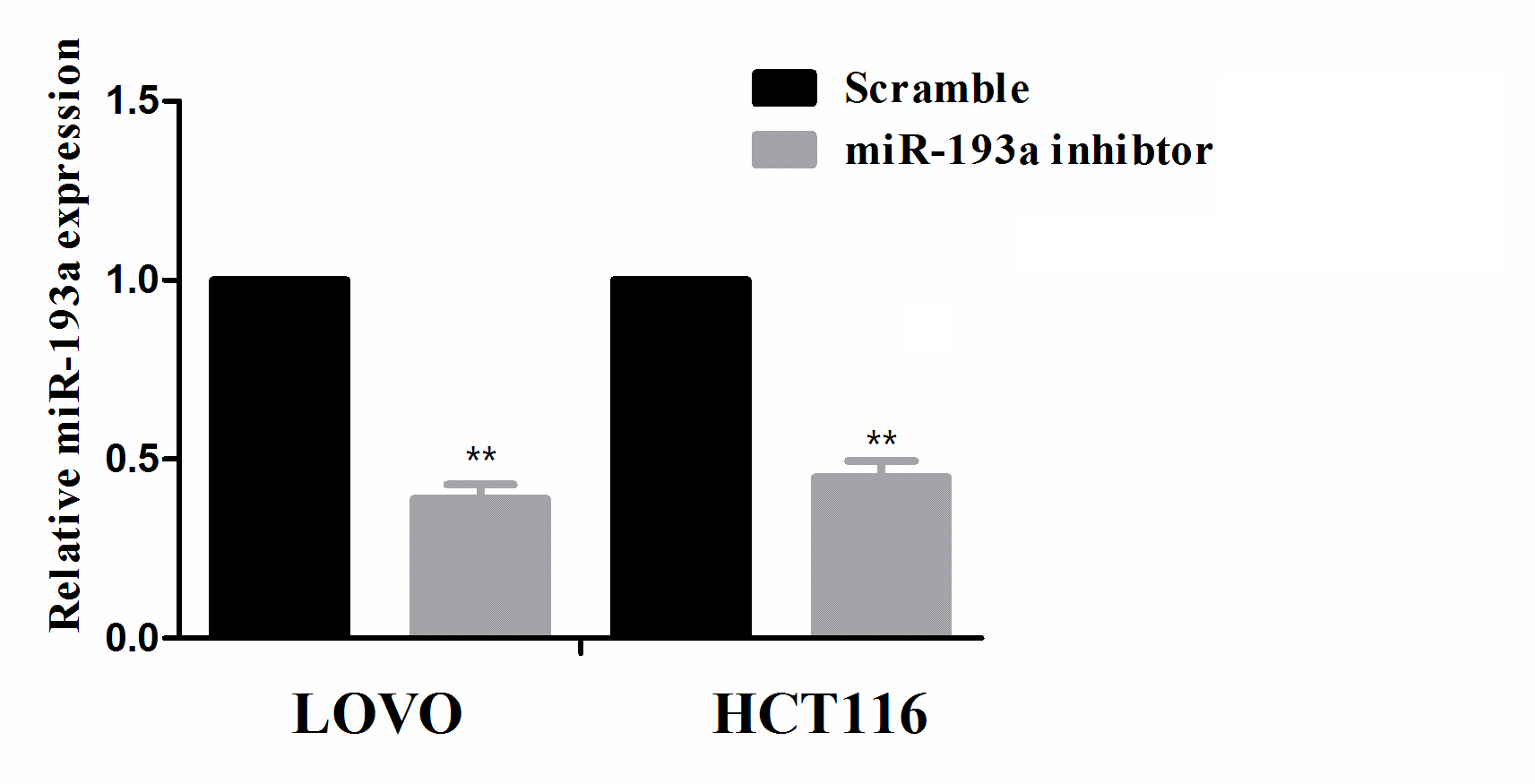

Supplement: Supplementary file 4 [file CPR-52-e12526-s004.tif]
