# Supplementary material for: LncRNA NEAT1 promotes the tumorigenesis of colorectal cancer by sponging miR‐193a‐3p
Source: Cell Prolif. 2018 Nov 8;52(1):e12526. doi: 10.1111/cpr.12526 (PMC6430453; doi:10.1111/cpr.12526)
Supplement: Supplementary file 5 [file CPR-52-e12526-s005.docx]

**Supplementary Tables**

**Supplementary Table 1** Clinicopathologic characteristics of CRC patients

| **Factors** | **Number (392, 100%)** |
| --- | --- |
| *Age (years)* |  |
| ≥60 | 256 (65.3%) |
| <60 | 136 (34.7%) |
| *Gender* |  |
| Male | 211 (53.8%) |
| Female | 181 (46.2%) |
| *Pathological stage* |  |
| Ⅰ/Ⅱ | 224 (57.1%) |
| Ⅲ/Ⅳ | 168 (42.9%) |
| *Localization* |  |
| Colon | 299 (76.3%) |
| Rectum | 93 (23.7%) |
| *TNM stage* |  |
| T1+T2 | 74 (18.9%) |
| T3+T4 | 318 (81.1%) |
| *Lymphatic invasion* |  |
| Negative | 285 (72.7%) |
| Positive | 107 (27.3%) |

**Supplementary Table 2** List of primers

| Gene | Sense sequence | Antisense sequence |
| --- | --- | --- |
| NEAT1 | 5’CTTCCTCCCTTTAAC  TTATCCATTCAC-3’ | 5’-CTCTTCCTCCACCA  TTACCAACAATAC-3’ |
| GAPDH | 5’ATCAGCAATGCCTCCTGCAC3’ | 5’ATGGCATGGACTGTGGTCAT 3’ |
| miR-193a-3p | 5'AACTGGCCTACAAAGTCCCAGT3' | provided in the miScript SYBR Green PCR kit. |
| U6 | 5'-CTCGCTTCGGCAGCACA-3' | 5'-CTCGCTTCGGCAGCACA-3' |

**Supplementary Table 3 Cox regression analysis of NEAT1 expression as OS predictor**

**for CRC patients**

| Variables | Univariate Cox regression analysis | |  | Multivariate Cox regression analysis | |
| --- | --- | --- | --- | --- | --- |
|  | RR (95% CI) | *P* value |  | RR (95% CI) | *P* value |
| *Age (years)* |  |  |  |  |  |
| <60 vs. ≥60 | 1.873 (1.029 to 2.719) | 0.038 |  | 1.662 (1.022 to 2.702) | 0.040 |
| *Gender* |  |  |  |  |  |
| Male vs. Female | 1.140 (0.758 to 1.714) | 0.528 |  | NA | NA |
| *Pathological stage* |  |  |  |  |  |
| Ⅰ/Ⅱ vs. Ⅲ/Ⅳ | 1.360 (0.907 to 2.040) | 0.137 |  | NA | NA |
| *Localization* |  |  |  |  |  |
| Colon vs. Rectum | 1.095 (0.684 to 1.754) | 0.705 |  | NA | NA |
| *TNM staging* |  |  |  |  |  |
| T1+T2 vs. T3+T4 | 1.033 (0.594 to 1.796) | 0.909 |  | NA | NA |
| *Lymphatic invasion* |  |  |  |  |  |
| Positive vs. Negative | 0.800 (0.503 to 1.272) | 0.345 |  | NA | NA |
| *NEAT1 expression* |  |  |  |  |  |
| High VS. Low | 1.209 (0.974 to 1.500) | 0.025 |  | 1.207 (0.971 to 1.500) | 0.091 |

**Supplementary Table 4** Cox regression analysis of NEAT1 expression as recurrence

predictor for CRC patients

| Variables | Univariate Cox regression analysis | |  | Multivariate Cox regression analysis | |
| --- | --- | --- | --- | --- | --- |
|  | RR (95% CI) | *P* value |  | RR (95% CI) | *P* value |
| *Age (years)* |  |  |  |  |  |
| <60 vs. ≥60 | 0.687 (0.443 to 1.066) | 0.094 |  | NA | NA |
| *Gender* |  |  |  |  |  |
| Male vs. Female | 1.459 (0.937 to 2.272) | 0.095 |  | NA | NA |
| *Pathological stage* |  |  |  |  |  |
| Ⅰ/Ⅱ vs. Ⅲ/Ⅳ | 1.176 (0.763 to 1.812) | 0.462 |  | NA | NA |
| *Localization* |  |  |  |  |  |
| Colon vs. Rectum | 0.914 (0.564 to 1.479) | 0.713 |  | NA | NA |
| *TNM staging* |  |  |  |  |  |
| T1+T2 vs. T3+T4 | 0.742 (0.427 to 1.289) | 0.289 |  | NA | NA |
| *Lymphatic invasion* |  |  |  |  |  |
| Positive vs. Negative | 1.042 (0.654 to 1.661) | 0.862 |  | NA | NA |
| *NEAT1 expression* |  |  |  |  |  |
| High VS. Low | 1.246 (1.001 to 1.550) | 0.042 |  | 1.246 (1.001 to 1.550) | 0.042 |

**Supplementary Table 5** The miRNAs were identified sponged by lncRNA_NEAT1 in cancer

| miRNA name | mirAccession | geneName | targetSites | bioComplex | clipReadNum | cancerNum |
| --- | --- | --- | --- | --- | --- | --- |
| hsa-miR-320b | MIMAT0005792 | NEAT1 | 2 | 7 | 5 | 3 |
| hsa-miR-107 | MIMAT0000104 | NEAT1 | 1 | 6 | 0 | 4 |
| hsa-miR-146b-5p | MIMAT0002809 | NEAT1 | 1 | 6 | 0 | 3 |
| hsa-miR-320d | MIMAT0006764 | NEAT1 | 2 | 7 | 5 | 6 |
| hsa-miR-433-3p | MIMAT0001627 | NEAT1 | 1 | 6 | 0 | 6 |
| hsa-miR-370-3p | MIMAT0000722 | NEAT1 | 2 | 8 | 4137 | 6 |
| hsa-miR-329-3p | MIMAT0001629 | NEAT1 | 2 | 7 | 5 | 3 |
| hsa-miR-495-3p | MIMAT0002817 | NEAT1 | 1 | 7 | 1977 | 4 |
| hsa-miR-365a-3p | MIMAT0000710 | NEAT1 | 1 | 8 | 6328 | 3 |
| hsa-miR-324-5p | MIMAT0000761 | NEAT1 | 2 | 6 | 0 | 4 |
| hsa-miR-193a-3p | MIMAT0000459 | NEAT1 | 1 | 7 | 2892 | 3 |
| hsa-miR-27a-3p | MIMAT0000084 | NEAT1 | 1 | 7 | 1722 | 3 |
| hsa-miR-181d-5p | MIMAT0002821 | NEAT1 | 2 | 8 | 4562 | 3 |
| hsa-miR-499a-5p | MIMAT0002870 | NEAT1 | 1 | 9 | 30 | 3 |
| hsa-miR-124-3p | MIMAT0000422 | NEAT1 | 2 | 9 | 4522 | 4 |
| hsa-miR-335-5p | MIMAT0000765 | NEAT1 | 1 | 6 | 0 | 5 |
| hsa-miR-320a | MIMAT0000510 | NEAT1 | 2 | 7 | 5 | 3 |
| hsa-miR-204-5p | MIMAT0000265 | NEAT1 | 2 | 7 | 4397 | 3 |
| hsa-miR-27b-3p | MIMAT0000419 | NEAT1 | 1 | 7 | 1722 | 3 |
| hsa-miR-503-5p | MIMAT0002874 | NEAT1 | 1 | 6 | 0 | 6 |
| hsa-miR-504-5p | MIMAT0002875 | NEAT1 | 1 | 6 | 0 | 3 |

**Supplementary Table 6** miRcode identification of the miRNAs binding to lncRNA_NEAT1

| microRNA family | Seed position | Seed type | Transcript region | Conservation | |
| --- | --- | --- | --- | --- | --- |
|  |  |  |  | Primates | Mammals |
| miR-193/193b/193a-3p | chr11:65191486 | 7-mer-m8 | ncRNA | 67% | 52% |
| miR-218/218a | chr11:65193974 | 7-mer-A1 | ncRNA | 89% | 57% |
| miR-103a/107/107ab | chr11:65191803 | 8-mer | ncRNA | 67% | 57% |

**Supplementary Table 7** Identification of the target genes of miR-193a-3p by TargetScan

| Ortholog of target gene | Representative transcript | 3P-seq tags + 5 | Cumulative weighted context++ score |
| --- | --- | --- | --- |
| IL17RD | ENST00000296318.7 | 108 | -0.95 |
| FLI1 | ENST00000527786.2 | 116 | -0.92 |
| SLC39A5 | ENST00000454355.2 | 1510 | -0.76 |
| ANAPC15 | ENST00000543587.1 | 36 | -0.73 |
| DCAF7 | ENST00000310827.4 | 3289 | -0.72 |
| AP2M1 | ENST00000382456.3 | 10753 | -0.7 |
| FAM84A | ENST00000295092.2 | 69 | -0.68 |
| SRSF2 | ENST00000392485.2 | 9241 | -0.67 |
| FHDC1 | ENST00000260008.3 | 53 | -0.67 |
| CBX7 | ENST00000216133.5 | 5 | -0.63 |
| SLC15A1 | ENST00000376503.5 | 15 | -0.61 |
| SLC16A6 | ENST00000327268.4 | 17 | -0.61 |
| ERBB4 | ENST00000342788.4 | 27 | -0.61 |
| DOK6 | ENST00000382713.5 | 8 | -0.6 |
| HELZ | ENST00000358691.5 | 571 | -0.6 |
| ST6GALNAC5 | ENST00000477717.1 | 7 | -0.6 |
| HOXD13 | ENST00000392539.3 | 25 | -0.59 |
| MSANTD2 | ENST00000239614.4 | 50 | -0.58 |
| ZNF385B | ENST00000410066.1 | 17 | -0.58 |
